# Products of Sulfide/Selenite Interaction Possess Antioxidant Properties, Scavenge Superoxide-Derived Radicals, React with DNA, and Modulate Blood Pressure and Tension of Isolated Thoracic Aorta

**DOI:** 10.1155/2019/9847650

**Published:** 2019-11-25

**Authors:** Marian Grman, Anton Misak, Lucia Kurakova, Vlasta Brezova, Sona Cacanyiova, Andrea Berenyiova, Peter Balis, Lenka Tomasova, Ammar Kharma, Enrique Domínguez-Álvarez, Miroslav Chovanec, Karol Ondrias

**Affiliations:** ^1^Institute of Clinical and Translational Research, Biomedical Research Center, Slovak Academy of Sciences, Dubravska cesta 9, 845 05 Bratislava, Slovakia; ^2^Department of Pharmacology and Toxicology, Faculty of Pharmacy, Comenius University, Odbojarov 10, 832 32 Bratislava, Slovakia; ^3^Faculty of Chemical and Food Technology, Slovak University of Technology, Radlinskeho 9, 812 37 Bratislava, Slovakia; ^4^Institute of Normal and Pathological Physiology, Centre of Experimental Medicine, Slovak Academy of Sciences, Dubravska cesta 9, 841 04 Bratislava, Slovakia; ^5^Instituto de Química Orgánica General, Consejo Superior de Investigaciones Científicas (IQOG-CSIC), 28006 Madrid, Spain; ^6^Cancer Research Institute, Biomedical Research Center, Slovak Academy of Sciences, Dubravska cesta 9, 845 05 Bratislava, Slovakia

## Abstract

Selenium (Se), an essential trace element, and hydrogen sulfide (H_2_S), an endogenously produced signalling molecule, affect many physiological and pathological processes. However, the biological effects of their mutual interaction have not yet been investigated. Herein, we have studied the biological and antioxidant effects of the products of the H_2_S (Na_2_S)/selenite (Na_2_SeO_3_) interaction. As detected by the UV-VIS and EPR spectroscopy, the product(s) of the H_2_S-Na_2_SeO_3_ and H_2_S-SeCl_4_ interaction scavenged superoxide-derived radicals and reduced ^·^cPTIO radical depending on the molar ratio and the preincubation time of the applied interaction mixture. The results confirmed that the transient species are formed rapidly during the interaction and exhibit a noteworthy biological activity. In contrast to H_2_S or selenite acting on their own, the H_2_S/selenite mixture cleaved DNA in a bell-shaped manner. Interestingly, selenite protected DNA from the cleavage induced by the products of H_2_S/H_2_O_2_ interaction. The relaxation effect of H_2_S on isolated thoracic aorta was eliminated when the H_2_S/selenite mixture was applied. The mixture inhibited the H_2_S biphasic effect on rat systolic and pulse blood pressure. The results point to the antioxidant properties of products of the H_2_S/selenite interaction and their effect to react with DNA and influence cardiovascular homeostasis. The effects of the products may contribute to explain some of the biological effects of H_2_S and/or selenite, and they may imply that a suitable H_2_S/selenite supplement might have a beneficial effect in pathological conditions arisen, e.g., from oxidative stress.

## 1. Introduction

Exogenously added and endogenously produced H_2_S affects many physiological and pathological processes [1–3]. Accumulating evidence supports the involvement of H_2_S in the regulation of cardiovascular homeostasis [4]. It has mostly beneficial effects during oxidative stress by reacting with reactive oxygen and nitrogen species, i.e., hydrogen peroxide (H_2_O_2_), superoxide anion radical (O_2_^·−^), hypochlorite (HOCl), or peroxynitrite (ONOO^−^) [5–8]. However, several effects of H_2_S on cells are toxic [9–11].

Selenium (Se) is a relatively rare but an essential trace element for humans, plants, and microorganisms. Se, which exerts multiple and complex effects on human health, is known as an antioxidant due to its presence in 25 selenoproteins in the form of selenocysteine amino acid. Both beneficial and detrimental effects of Se deficiency and/or supplementation are well known. The biological effects of Se compounds (selenite, selenate, selenocysteine, and selenomethionine) on cardiac oxidative damage, heart disease, cancer prevention, immunity, diabetes, neuroregeneration, or dementia have been reported [12–17]. However, the beneficial effect of Se supplementation for men's health is still a controversial issue [18–23]. Selenite, which is a common Se supplement, is considered as a promising anticarcinogen [24–26]. It can induce apoptosis in cancer cells through the production of reactive oxygen species (ROS) leading to oxidative stress [27, 28]. However, Se compounds were also found to damage DNA in healthy cells [29] and therefore may not be considered as a suitable protective agent against cancer and/or other chronic diseases. Actually, they can cause or advance some kinds of cancers [30, 31]. The exact mechanisms of the beneficial and toxic effects of Se are not yet fully understood, giving rise to further uncertainty about its potential use in nutrition supplements and/or clinical treatment.

Se and H_2_S are present in living organisms, and each one has beneficial and/or toxic effects through its interaction mostly with ROS [1, 2, 30–33]. However, the biological effects of products of the H_2_S/selenite interaction are not yet known, namely, their involvement in the production and/or inhibition of ROS, reaction with DNA, or influence on cardiovascular system. Therefore, we have studied the effects of products of the H_2_S/selenite interaction on O_2_^·−^ and ^**·**^cPTIO radicals, DNA cleavage, tension of isolated aortic rings, and rat blood pressure (BP). We found that the products have significant biological effects that differ from those caused by H_2_S or by selenite on their own. These results may contribute to the understanding of possible coupled biological effects of H_2_S and Se.

## 2. Material and Methods

### 2.1. Chemicals

#### 2.1.1. Selenium Compounds

Stock solutions of sodium selenite (Na_2_SeO_3_, 10 or 40 mmol L^−1^, Merck K34993707-542 or Sigma 214485), selenium tetrachloride (SeCl_4_, 10 mmol L^−1^, Aldrich 323527), and sodium selenate (Na_2_SeO_4_, 10 mmol L^−1^, Sigma S0882) were prepared freshly in deionized H_2_O, stored at 23°C, and used within 5 h. Na_2_SeO_3_ dissociates in solution to yield mostly H_2_SeO_3_ at acidic pH, HSeO_3_^−^ at neutral pH, and SeO_3_^2−^ at alkaline pH. For simplicity, the term SeO_3_^2−^ is employed as representative expression to encompass the total mixture of these different (de)protonation states.

#### 2.1.2. Hydrogen Peroxide

Hydrogen peroxide (H_2_O_2_) (14.7 mol L^−1^; Sigma-Aldrich 85321), according to a particular experiment, was diluted in H_2_O or in 100 mmol L^−1^ sodium phosphate buffer, supplemented with 200 or 50 *μ*mol L^−1^ DTPA, pH 7.4, 37°C before application.

#### 2.1.3. Radicals

5-*tert*-butoxycarbonyl-5-methyl-1-pyrroline-*N*-oxide (BMPO, 100 mmol L^−1^, Dojindo B568-10, Japan) was dissolved in deionized H_2_O, stored at -80°C, and used after thawing. 2-(4-Carboxyphenyl)-4,4,5,5-tetramethylimidazoline-1-oxyl-3-oxide (^·^cPTIO, 10 mmol L^–1^, Cayman 81540 or Sigma C221) was dissolved in deionized H_2_O and was stored at -20°C for several weeks.

#### 2.1.4. Sulfide

Na_2_S (100 mmol L^−1^ stock solution, Dojindo SB01, Japan) was dissolved in argon degassed deionized H_2_O, stored at −80°C, and used immediately after thawing. Na_2_S dissociates in aqueous solution and reacts with H^+^ to yield H_2_S, HS^−^, and a trace of S^2−^. We use the term H_2_S to describe the total mixture of H_2_S, HS^−^, and S^2−^ forms. The stock concentration was checked by UV-VIS spectroscopy: by the absorbance of 1000x diluted stock solution at 230 nm (*ε*_230 nm_ = 7700 mol^−1^ L cm^−1^, diluted by deionized water) and also by the reduction of 100 *μ*mol L^−1^ DTNB by 2000x diluted stock solution (1 H_2_S molecule generates 2 TNB^−^ equivalents, *ε*_412 nm_ = 14,100 mol^−1^ L cm^−1^, measured in 1 mmol L^−1^ phosphate buffer), according to Nagy et al. [34].

#### 2.1.5. Buffers

100 mmol L^−1^ sodium phosphate buffer supplemented with 100 *μ*mol L^−1^ diethylenetriaminepentaacetic acid (DTPA), pH 6.5, 7.0, 7.4, 8.0, and 9.0, 37°C, was employed for UV-VIS experiments. 50 and 25 mmol L^−1^ sodium phosphate buffer, supplemented with 100 and 50 *μ*mol L^−1^ DTPA, pH 7.4, 37°C, was used for electron paramagnetic resonance (EPR) and plasmid DNA (pDNA) cleavage studies, respectively.

### 2.2. UV-VIS of ^·^cPTIO

To obtain 1 mL of the working solution, 10 or 100 *μ*L of stock solution of the compounds studied was added to the appropriate volume (990 or 900 *μ*L, respectively) of 100 mmol L^−1^ sodium phosphate buffer (at given pH, 37°C) containing the final concentrations of 100 *μ*mol L^−1 **·**^cPTIO and 100 *μ*mol L^−1^ DTPA. UV-VIS absorption spectra (900-190 nm) were recorded every 30 s for 20 to 40 min with a Shimadzu 1800 (Kyoto, Japan) spectrometer at 37°C. The ^**·**^cPTIO extinction coefficient of 920 mol^−1^ L cm^−1^ at 560 nm was used. The reduction of the ^**·**^cPTIO radical was determined as the decrease of the absorbance at 560 nm (absorption maximum of ^**·**^cPTIO in VIS range) or at 358 nm after subtracting the absorbances at 730 or at 420 nm, respectively [5, 35].

To study the involvement of O_2_ in the H_2_S/SeO_3_^2−^-induced reduction of the ^·^cPTIO radical, 10 mmol L^−1^ Na_2_S in H_2_O, 10 mmol L^−1^ Na_2_SeO_3_ in H_2_O, and 102 *μ*mol L^−1 ·^cPTIO in the 100 mmol L^–1^ sodium phosphate buffer, supplemented with 100 *μ*mol L^−1^ DTPA (pH 7.4; 37°C), were deaerated with argon for 10 min at 37°C. The compounds were mixed in a closed UV-cuvette, and the UV-VIS spectra were recorded. The O_2_ concentration in the deaerated samples was 3-5%, confirmed with an oxygen electrode (OXELP, SYS-ISO2, WPI, USA). In all UV-VIS experiments, H_2_O was used as a blank.

### 2.3. EPR of the ^·^BMPO Adducts

To study the ability of H_2_S/SeO_3_^2−^ to scavenge the O_2_^·−^ radical or its derivatives produced in DMSO/KO_2_ solution, sample preparation and EPR measurements were conducted in accordance with previously reported protocols [5]. The solution (final concentrations) of BMPO (20 mmol L^−1^), DTPA (100 *μ*mol L^−1^) in sodium phosphate buffer (50 mmol L^−1^, pH 7.4) was incubated for 1 min at 37°C; an aliquot of the compound was added, followed by saturated KO_2_/DMSO solution (10% *v*/*v* DMSO/final buffer) 3 s later. The sample was mixed for 5 s and transferred to a standard cavity aqueous EPR flat cell. The first EPR spectrum was recorded 2 min after the addition of KO_2_/DMSO solution at 37°C. The sets of individual EPR spectra of the ^·^BMPO spin adducts were recorded as 15 sequential scans, each 42 s, with a total time of 11 min. Each experiment was repeated at least twice. EPR spectra of the ^·^BMPO spin adducts were measured on a Bruker EMX spectrometer, X-band ~9.4 GHz, 335.15 mT central field, 8 mT scan range, 20 mW microwave power, 0.1 or 0.15 mT modulation amplitude, 42 s sweep time, 20.48 ms time constant, and 20.48 ms conversion time at 37°C. Intensities of the ^·^BMPO adducts in the EPR spectra were reproducible, when the KO_2_/DMSO stock solution was stored at 5°C for 1 day or at 23 ± 1°C for 4 h.

### 2.4. Plasmid DNA Cleavage

pDNA cleavage assay with the use of pBR322 plasmid (New England BioLabs Inc., N3033L) was performed as reported previously [5, 36]. In this assay, all samples contained 0.2 *μ*g pDNA in sodium phosphate buffer (25 mmol L^−1^ sodium phosphate, 50 *μ*mol L^−1^ DTPA, pH 7.4, 37°C). After addition of compounds, the resulting mixtures were incubated for 30 min at 37°C. All concentrations listed in the section were final in the samples. After incubation, the reaction mixtures were subjected to 0.6% agarose gel electrophoresis. Samples were electrophoresed in TBE buffer (89 mmol L^−1^ Tris, 89 mmol L^−1^ boric acid, and 2 mmol L^−1^ EDTA) at 5.5 V cm^−1^ for 2 h; gels were stained with GelRed™ Nucleic Acid Gel Stain and photographed using a UV transilluminator. Integrated densities of all pBR322 forms in each lane were quantified using the TotalLab TL100 image analysis software to estimate pDNA cleavage efficiency (Nonlinear Dynamic Ltd., USA).

### 2.5. Guide for the Use and Care of Laboratory Animals

#### 2.5.1. Isolated Thoracic Aorta

Procedures were performed in accordance with the Institutional Guidelines of the Ethical Committee on the Ethics of Procedures in Animal, Clinical and other Biomedical Experiments (permit number: EC/CEM/2017/4) of the Institute of Normal and Pathological Physiology, Centre of Experimental Medicine and were approved by the State Veterinary and Food Administration of the Slovak Republic and by an Ethical Committee according to the European Convention for the Protection of Vertebrate Animals used for Experimental and other Scientific Purposes, Directive 2010/63/EU of the European Parliament. The Institute of Normal and Pathological Physiology provided veterinary care.

#### 2.5.2. Rat Blood Pressure

All procedures were approved by the State Veterinary and Food Administration of the Slovak Republic (No.: Ro-1545/15-221) according to the guidelines from Directive 2010/63/EU of the European Parliament. Experiments were carried out according to the guidelines laid down by the animal welfare committee of the Institute of Normal and Pathological Physiology of the Slovak Academy of Sciences and conformed to the principles and regulations, as described in the editorial by Grundy [37].

### 2.6. Functional Study of Isolated Thoracic Aorta

Normotensive Wistar Kyoto (WKY) rats (307 ± 4.3 *g*) were killed by decapitation after a brief anesthetization with CO_2_, and the thoracic aorta was isolated as described in our previous study [38]. The changes in isometric tension were measured by the electromechanical transducers (FSG-01, MDE, Budapest, Hungary). The resting tension of 1 g was applied to each ring and maintained throughout a 45 to 60 min of equilibration period until stress relaxation no longer occurred. Changes in thoracic aorta tension were followed by noradrenaline (NA; 1 *μ*mol L^−1^) precontracted arterial rings after a stable plateau was achieved.

### 2.7. Functional Study of Rat Blood Pressure

Male Wistar rats (*n* = 10; 350 ± 40 g) were from the Department of Toxicology and Laboratory Animal Breeding at Dobra Voda, Slovak Academy of Sciences, Slovakia. The rats were housed under a 12 h light-12 h dark cycle, at a constant humidity (45-65%) and temperature (20-22°C), with free access to standard laboratory rat chow and drinking water. The *Institute of Experimental Pharmacology* and *Toxicology*, Centre of Experimental Medicine, Slovak Academy of Sciences, provided veterinary care. The tranqualizer xylazine (Rometar) was purchased from Zentiva (Czech Republic), and the anesthetic combination of tiletamine+zolazepam (Zoletil 100) was acquired from Virbac (France). All other chemicals were purchased from Sigma-Aldrich. Experiments were carried out as previously described [39]. Rats were anesthetized with Zoletil 100 (tiletamine+zolazepam, 80 mg kg^−1^, i.p.) and Rometar (xylazine, 5 mg kg^−1^, i.p.). During the anesthesia, BP, heart rate, and reflex responses to mechanical stimuli were monitored. The animals were under anesthesia during the whole experiment and were euthanized with an overdose of Zoletil *via* jugular vein at the end of the surgical procedure. All experiments were supervised and performed under the same experimental conditions.

### 2.8. Blood Pressure Measurement

The right jugular vein was cannulated to administer compounds under anesthesia as described above. The left arteria carotis communis was cannulated for inserting the fiber optic microcatheter pressure transducers (FISO LS 2F Harvard Apparatus, USA). The analog signal was digitalized at 10 kHz, filtered at 1 kHz, and recorded by DEWESoft 6.6.7 (GmbH, Austria). The signal was evaluated 5 s before and 10 min after compound administration. After stabilization of BP (10-20 min), the compounds were administered into the right jugular vein as a bolus of 500 *μ*L kg^−1^ over 15 s period. The solution of the H_2_S/SeO_3_^2−^ mixture (10/5 in mmol L^−1^) was prepared as follows: to 123.5 *μ*L of 100 mmol L^−1^ phosphate buffer, 100 *μ*mol L^−1^ DTPA, 14 *μ*L of 1 mol L^−1^ HCl was added, followed by 62.5 *μ*L of 40 mmol L^−1^ Na_2_SeO_3_ in 0.9% NaCl, and finally 50 *μ*L of 100 mmol L^−1^ Na_2_S in H_2_O was added. The pH of the buffered mixture was 7.4. The mixture was incubated for 40 ± 10 s at 23°C before i.v. administration. Unbuffered H_2_S/SeO_3_^2−^ mixture (10/5 in mmol L^−1^) was prepared, when 0.9% NaCl was used instead of phosphate buffer and HCl. The pH of the unbuffered mixture was ~11 measured by a pH paper indicator.

### 2.9. Statistical Analysis

Unless otherwise stated, data are represented as the means ± S.E.M. Statistical significance was determined by Student's *t*-test or one-way ANOVA followed by the multiple comparison test. Differences between means were considered significant at ^∗^*P* ≤ 0.05. Data analysis and plot construction were carried out using SigmaPlot 12 (Systat Software GmbH).

## 3. Results

### 3.1. H_2_S Interacts with Na_2_SeO_3_ and SeCl_4_, but Not with Na_2_SeO_4_, to Form Initial Reactive Intermediate(s), which Reduce the ^·^cPTIO Radical

**Figure 1:**
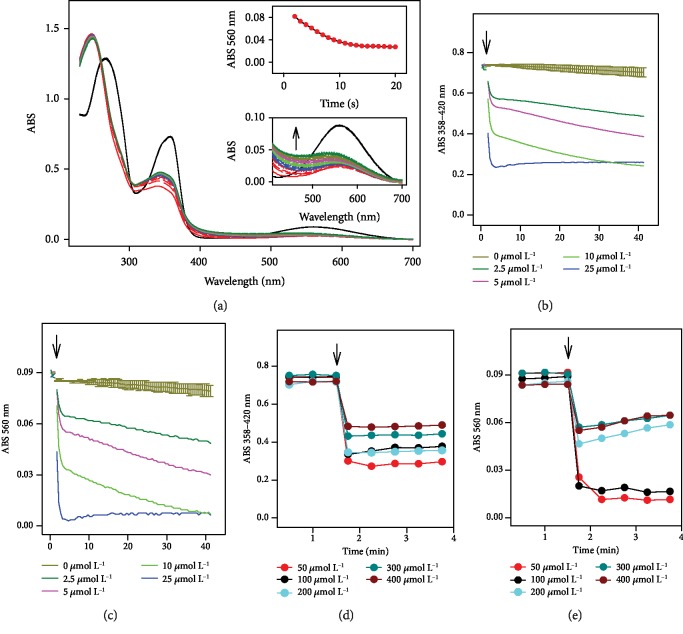
Interaction of ^·^cPTIO/SeO_3_^2−^ with H_2_S. (a) Time resolved UV-VIS spectra of the interaction of 100 *μ*mol L^−1 ·^cPTIO with 100 *μ*M SeO_3_^2−^ (3 times repeated every 30 s, black) and subsequent addition of 100 *μ*mol L^−1^ H_2_S. Spectra were collected every 30 s for 15 min; the first spectrum, indicated by the solid red line, was measured 15 s after addition of H_2_S. Top inset: kinetics of changes in absorbance at 560 nm after addition of 100 *μ*mol L^−1^ H_2_S into ^·^cPTIO/SeO_3_^2−^ (100/100 in *μ*mol L^−1^) solution at time 0 s. Bottom inset: details of the time resolved spectra of the ^·^cPTIO/SeO_3_^2−^ (100/100 in *μ*mol L^–1^) interaction before (black) and after addition of H_2_S (100 *μ*mol L^−1^, the first spectrum is indicated by the solid red line, which is followed each 30 s by: long dash red, medium dash red, short dash red, dotted red, solid blue line, long dash blue, medium dash blue, etc.). (b, c, d, e) H_2_S (100 *μ*mol L^−1^) was added to 100 *μ*mol L^−1^ solution of ^·^cPTIO containing different concentrations of SeO_3_^2−^ (0-400 *μ*mol L^−1^, see legend). The kinetics of the reduction of 100 *μ*mol L^−1 ·^cPTIO before and after addition of 100 *μ*mol L^−1^ H_2_S (marked by arrow) was monitored as a decrease of the absorbance at 358 nm minus the absorbance at 420 nm (b, d) and also as a decrease of the absorbance at 560 nm (c, e).

**Figure 2:**
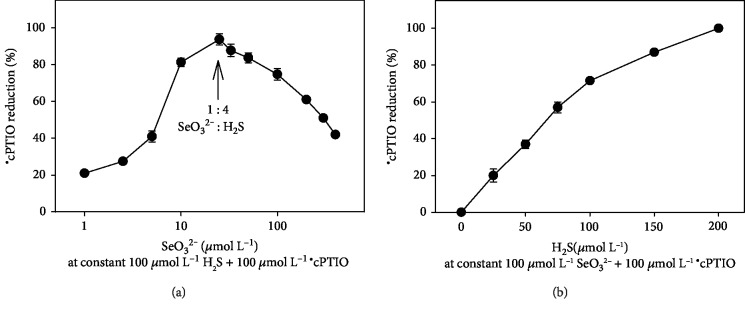
Effect of the SeO_3_^2−^ and H_2_S concentration on the reduction of ^·^cPTIO. (a) Effect of the SeO_3_^2−^ concentration (1-400 *μ*mol L^−1^) on the reduction of ^·^cPTIO (100 *μ*mol L^−1^) in the presence of H_2_S (100 *μ*mol L^−1^). Arrow indicates the 1 : 4 molar ratio of SeO_3_^2−^ : H_2_S. (b) Effect of the H_2_S concentration (0-200 *μ*mol L^−1^) on the reduction of ^·^cPTIO (100 *μ*mol L^−1^) in the presence of SeO_3_^2−^ (100 *μ*mol L^−1^). The reduction of ^·^cPTIO was evaluated as the decrease of the absorbance at 560 nm after 2.25 min of the reaction (pH 7.4, 37°C). All values represent the means ± S.E.M., *n* = 2‐4.

Since the antioxidant properties of H_2_S/SeO_3_^2−^ products are unknown, we have used the ^·^cPTIO radical to study the reducing properties of the products of H_2_S/SeO_3_^2−^ interaction. The ^·^cPTIO radical is stable in aqueous solution, and its formation and reduction can be monitored by the UV-VIS spectrophotometry at 358 or 560 nm. Even in the presence of up to 100 *μ*mol L^−1^ H_2_S or 100-400 *μ*mol L^−1^ SeO_3_^2−^, the absorbance (ABS) of the radical at 358 and 560 nm decreases only by <7% after 40 min, indicating that neither H_2_S nor SeO_3_^2−^ on its own reduces this radical (Figure 1). In contrast, once H_2_S (25-100 *μ*mol L^−1^) was added to the ^·^cPTIO/SeO_3_^2−^ (100/2.5-400 *μ*mol L^−1^) mixture, or SeO_3_^2−^ was added to ^·^cPTIO/H_2_S, the absorbances at 358 and 560 nm decreased rapidly over the time (≤30 s), indicating a possible formation of strong reducing agent(s) which fastly and efficiently reduced the ^·^cPTIO radical (Figures 1 and 2). Similar results were obtained when SeCl_4_, but not Na_2_SeO_4_, was used instead of SeO_3_^2−^ (Figures [Supplementary-material supplementary-material-1] and [Supplementary-material supplementary-material-1]).

**Figure 3:**
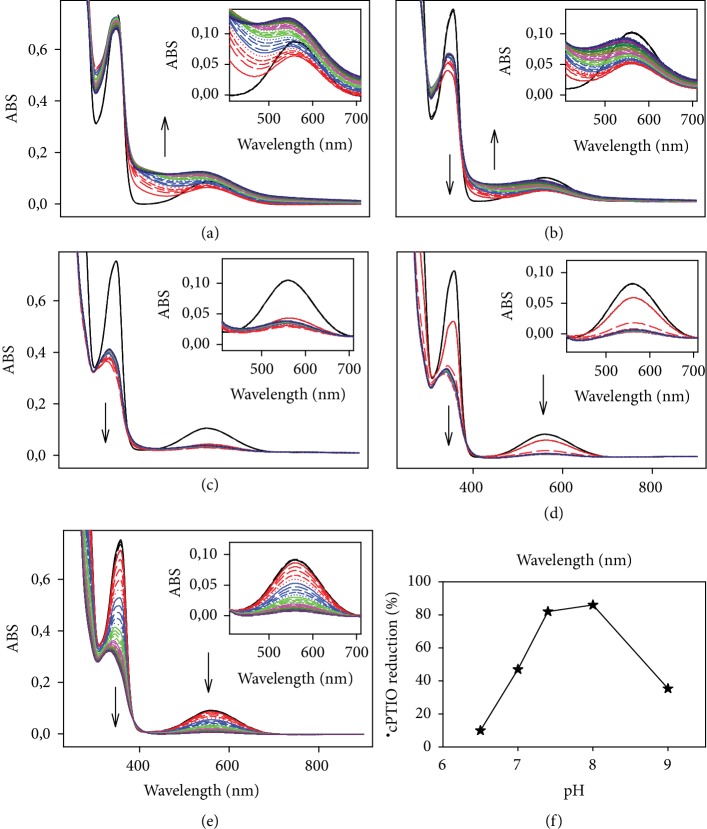
Effect of pH on the reduction of ^·^cPTIO in the presence of SeO_3_^2−^ induced by H_2_S. Time resolved UV-VIS spectra of the interaction of H_2_S (100 *μ*mol L^−1^ final) with ^·^cPTIO/SeO_3_^2−^ (100/50 in *μ*mol L^−1^ final) at pH 6.5 (a), 7.0 (b), 7.4 (c), 8.0 (d), and 9.0 (e). The first spectrum was recorded 15 s after H_2_S addition (solid red line) followed each 30 s by: long dash red, medium dash red, short dash red, dotted red, solid blue line, long dash blue, medium dash blue, etc. Samples were measured every 30 s for 20 min. The black line represents the spectrum of ^·^cPTIO/SeO_3_^2−^ before the H_2_S addition. (f) Dependence of ^·^cPTIO (100 *μ*mol L^−1^) reduction by H_2_S/SeO_3_^2−^ (100/50 in *μ*mol L^−1^) on pH. Data were taken from the spectra shown in (a), (b), (c), (d), and (e) as a minimum of A_560_ in the range of 0-4 min. The buffers consisted of 100 mmol L^−1^ sodium phosphate, 100 *μ*M DTPA, 37°C, and pH values were adjusted to desired pH.

The reduction of ^·^cPTIO followed a bell-shaped dependence on the concentration of SeO_3_^2−^ at a constant ^·^cPTIO/H_2_S concentration (Figure 2(a)), with a maximum radical scavenging activity at an H_2_S : SeO_3_^2−^ ratio of roughly 4 : 1. The ability of H_2_S to reduce ^·^cPTIO in the presence of SeO_3_^2−^ increased with the increasing H_2_S concentration (Figure 2(b)) and followed also a bell-shaped dependence on pH (Figures 3 and [Supplementary-material supplementary-material-1]).

**Figure 4:**
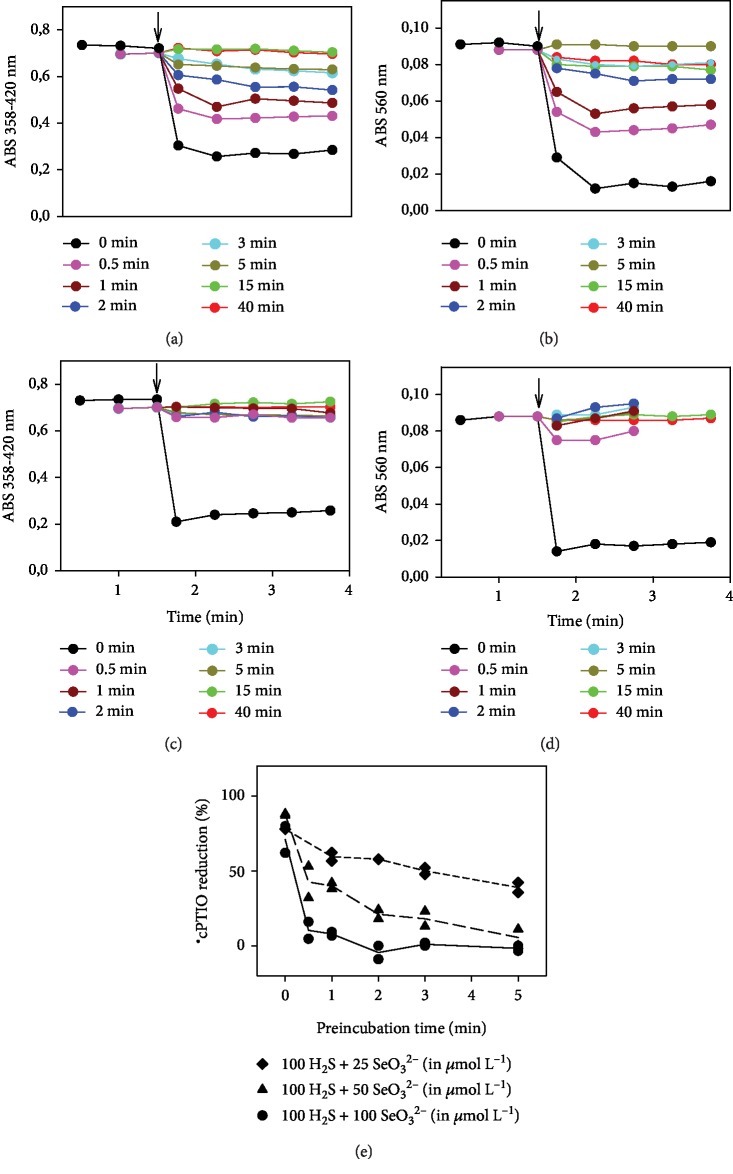
Influence of the preincubation time of the H_2_S/SeO_3_^2−^ mixture on the time-dependent reduction of ^·^cPTIO. The reduction of ^·^cPTIO (100 *μ*mol L^−1^) once added into the preincubated H_2_S/SeO_3_^2−^ mixture (100/50 in *μ*mol L^−1^, (a, b); 100/100 in *μ*mol L^−1^ (c, d)) was evaluated as the decrease of absorbance at 358 nm minus 420 nm (a, c) and the decrease of absorbance at 560 nm (b, d), respectively. The preincubation time of the H_2_S/SeO_3_^2−^ mixture was 0-40 min (see legend). (e) Impact of the preincubation time of the H_2_S/SeO_3_^2−^ mixture on the reduction of ^·^cPTIO. ^·^cPTIO (100 *μ*M) was added into preincubated H_2_S/SeO_3_^2−^ (in *μ*mol L^−1^, 100/100, circles; 100/50, triangles; 100/25, diamonds) at pH 7.4, 37°C. Data represent the reduction of ^·^cPTIO in 1^st^ minute after addition of ^·^cPTIO.

If H_2_S and SeO_3_^2−^ were preincubated for different periods of time before the addition to ^·^cPTIO, it clearly resulted in the highest radical scavenging activity, which was subsequently lost over the time (Figure 4). An H_2_S/SeO_3_^2−^ (100/100 in *μ*mol L^−1^) mixture preincubated for ≥1 min prior to ^·^cPTIO addition did not reduce ^·^cPTIO, demonstrating that later products of the reaction of sulfide with SeO_3_^2−^ could not be responsible for the reduction of the radical and that the relevant active species were formed swiftly, in less than 1 min and have a short lifetime, as recently suggested [40]. Notably, formation of these active early intermediates to reduce ^·^cPTIO was prolonged with the increase of the H_2_S/SeO_3_^2−^ ratio (Figure 4(e)). At H_2_S/SeO_3_^2−^ concentration of 100/25 in *μ*mol L^−1^ and 5 min preincubation time, the mixture still possessed around 50% potency to reduce ^·^cPTIO (Figure 4(e)). This timing, once more, accounts for a rapidly formed selenosulfide intermediate as beeing the ultimate responsible for this radical scavenging activity, a species also possibly being sensitive to oxidation over prolonged time periods (Figure 4).

While the available evidence is in accordance with the formation of HSSeSH as the main reactive species, there are other chalcogen-based candidates which are good reducing agents, namely, hydrogen selenide (H_2_Se), hydroselenide anion (HSe^−^), selenide (Se^2−^), persulfides, and polysulfides (S*_x_*^2–^) [41–43]. Interestingly, O_2_ does not seem to play a major role in the H_2_S/SeO_3_^2−^-induced reduction of the ^·^cPTIO radical probably due to slower kinetics of interaction of reactants and/or intermediates with O_2_ in comparison to the rate of ^·^cPTIO reduction [44–48]. Under argon flushed conditions, reduction of the ^·^cPTIO radical was neither enhanced nor suppressed significantly, hence ruling out any major involvement of H_2_Se, as this selenium compound is highly sensitive to O_2_ ([Supplementary-material supplementary-material-1]).

### 3.2. EPR of ^·^BMPO-OOH: The Initial Products of the H_2_S/SeO_3_^2−^ Interaction Also Scavenge Derivatives of Superoxide Anion (O_2_^·–^) Radical

We aimed to ascertain whether the initial products of the H_2_S/SeO_3_^2−^ interaction are able to scavenge other radicals, i.e., O_2_^·−^ or its derivatives. The interactions with O_2_^·−^ were studied with the EPR spin trap method based on the reaction of this dioxygen radical with BMPO to form the ^·^BMPO-OOH adduct [49]. This assay was chosen due to biological and mechanistical reason; O_2_^·−^ is a simple radical reduced by one-electron transfer.

**Figure 5:**
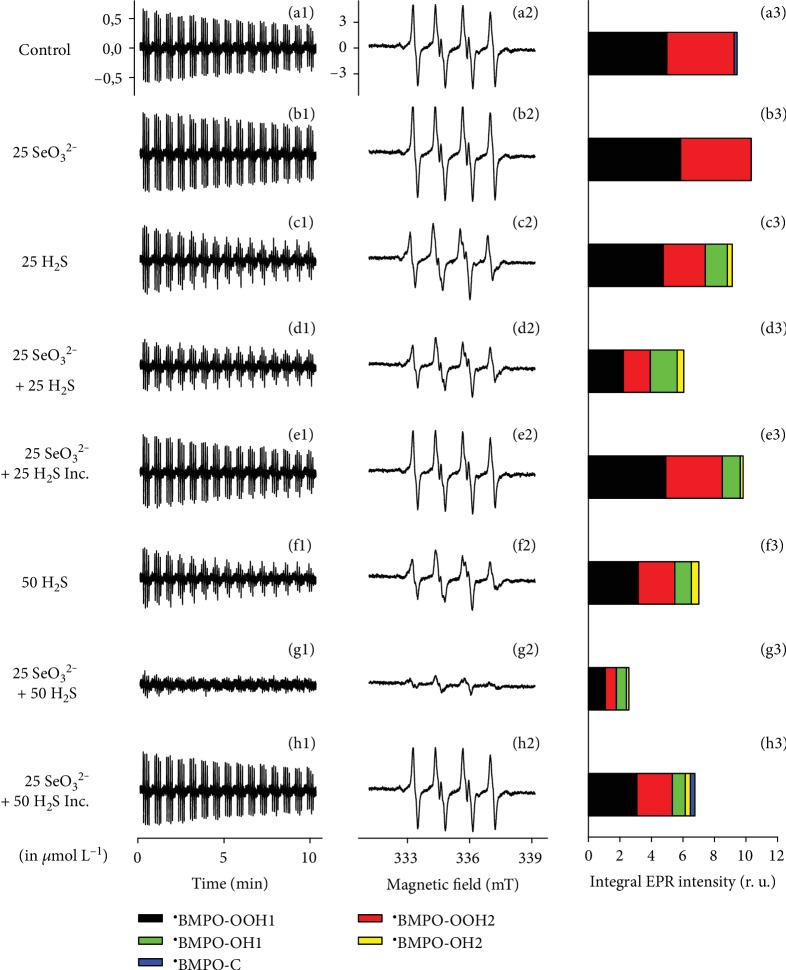
EPR spectra of ^·^BMPO in the presence of O_2_^·−^ are modulated by H_2_S/SeO_3_^2−^. Representative EPR spectra of the ^·^BMPO adducts were monitored in 10% *v*/*v* saturated KO_2_/DMSO solution in 50 mmol L^−1^ sodium phosphate buffer, 0.1 mmol L^−1^ DTPA, pH 7.4, 37°C in the presence of the various investigated chalcogen species and 20 mmol L^−1^ BMPO. Sets of individual EPR spectra of the ^·^BMPO adducts monitored upon 15 sequential scans, each 42 s (a1-h1), starting acquisition 2 min after sample preparation in: control 10% *v*/*v* KO_2_/DMSO in the buffer (a1), the KO_2_/DMSO in the presence of 25 *μ*mol L^−1^ SeO_3_^2−^ (b1), 25 *μ*mol L^−1^ H_2_S (c1), mixture of 25/25 in *μ*mol L^−1^ H_2_S/SeO_3_^2−^ (d1), 25/25 in *μ*mol L^−1^ H_2_S/SeO_3_^2−^ preincubated 5 min before KO_2_/DMSO stock solution addition (e1), 50 *μ*mol L^−1^ H_2_S (f1), mixture of 50/25 in *μ*mol L^−1^ H_2_S/SeO_3_^2−^ (g1), and 50/25 in *μ*mol L^−1^ H_2_S/SeO_3_^2−^ preincubated 5 min before addition of KO_2_/DMSO stock solution (h1). The spectra (a2-h2) show details of the accumulated first ten spectra of the (a1-h1) sets. The intensities of the time-dependent EPR spectra (a1-h1) and detailed spectra (a2-h2) are comparable; they were measured under identical EPR settings. EPR modulation amplitude 0.15 mT. (a3-h3) Comparison of the integral intensity of individual components of simulated BMPO+O_2_^·−^ without (control) and with chalcogen species shown in (a1-h1). The first five EPR spectra were accumulated and used for simulation. The data represent the means of *n* = 2; standard error was ≤10% of the mean value. Simulated relative intensities of the two conformers of the radicals: ^·^BMPO-OOH1 (black), ^·^BMPO-OOH2 (red), ^·^BMPO-OH1 (green), ^·^BMPO-OH2 (yellow), and ^·^BMPO-C (blue).

O_2_^·−^ was dissolved in phosphate buffer (pH 7.4, 37°C) and trapped by BMPO. Under these conditions, the relative intensity of the ^·^BMPO-OOH adduct decreased slowly over the time and was comparable to the values reported under physiological conditions (Figures 5(a1)–5(a3)) [49]. The addition of SeO_3_^2−^ (25 and 50 *μ*mol L^–1^) did not significantly interfere with the ^·^BMPO-OOH adduct formation, its concentration, and rate of decay (Figures 5(b1)–5(b3), [Supplementary-material supplementary-material-1]). In contrast, H_2_S (25 and 50 *μ*mol L^−1^) decreased the ^·^BMPO-OOH concentration and increased the rate of the decay, and this “antioxidant” activity of H_2_S was increased further in the presence of SeO_3_^2−^ (more than three times at a 50/25 in *μ*mol L^−1^ H_2_S/SeO_3_^2−^ ratio). This indicates that H_2_S/SeO_3_^2−^ products scavenge O_2_^·−^ and/or its derivatives. The effects of H_2_S/SeO_3_^2−^ were less pronounced when KO_2_/DMSO was added 5 min after addition of H_2_S/SeO_3_^2−^, suggesting that the reducing intermediate had already decomposed at this stage (Figure 5, [Supplementary-material supplementary-material-1]). Similar results were obtained when H_2_S was incubated with SeCl_4_ ([Supplementary-material supplementary-material-1]). This indicates that the production of highly active species, products of the H_2_S/SeO_3_^2−^ interaction, was time-dependent: they appeared within a few seconds after addition of SeO_3_^2−^ to the H_2_S solution and their effects diminished after few minutes of interaction.

From our previous studies of O_2_^·−^ reaction with BMPO [5], we assumed that the EPR spectra of the ^·^BMPO adducts were superposed on the ^·^BMPO-OOH/OH radicals with minor contribution from the ^·^BMPO-C radical. Therefore, we simulated the spectra using hyperfine coupling constants for ^·^BMPO-OOH, ^·^BMPO-OH, and ^·^BMPO-C (derived from DMSO) radicals. The means of hyperfine coupling constants used were as follows: ^·^BMPO-OOH1 (black) *a*_*N*_ = 13.30 ± 0.03 *G*, *a*_*H*_ = 11.7 ± 0.1 *G*; ^·^BMPO-OOH2 (red) *a*_*N*_ = 13.24 ± 0.03 *G*, *a*_*H*_ = 9.4 ± 0.1 *G*; ^·^BMPO-OH1 (green) *a*_*N*_ = 13.7 ± 0.3 *G*, *a*_*H*_ = 12.3 ± 0.4 *G*; ^·^BMPO-OH2 (yellow) *a*_*N*_ = 13.6 ± 0.2 *G*, *a*_*H*_ = 15.3 ± 0.1 *G*; and ^·^BMPO-C (blue) *a*_*N*_ = 15.2 ± 0.1 *G*, *a*_*H*_ = 21.5 ± 0.1 *G*. The constants are similar to those reported by Zhao et al. [49]. The simulation revealed that O_2_^·−^ was trapped in the control and in the presence of SeO_3_^2−^ (Figures 5(a) and 5(b)), since the EPR spectra of ^·^BMPO-OOH were only observed. However, ^·^BMPO-OH component was present in the samples containing H_2_S alone or with SeO_3_^2−^ (Figures 5(c)–5(h)). The results may indicate that H_2_S alone or with SeO_3_^2−^ decomposed ^·^BMPO-OOH to ^·^BMPO-OH and/or ^·^OH was formed as a result of compound presence. Since the first spectrum was recorded 110 ± 15 *s* after sample preparation, we cannot exclude a possibility of trapping of other radicals with lifetimes shorter than 110 s.

### 3.3. H_2_S/SeO_3_^2−^ Cleaves pDNA

**Figure 6:**
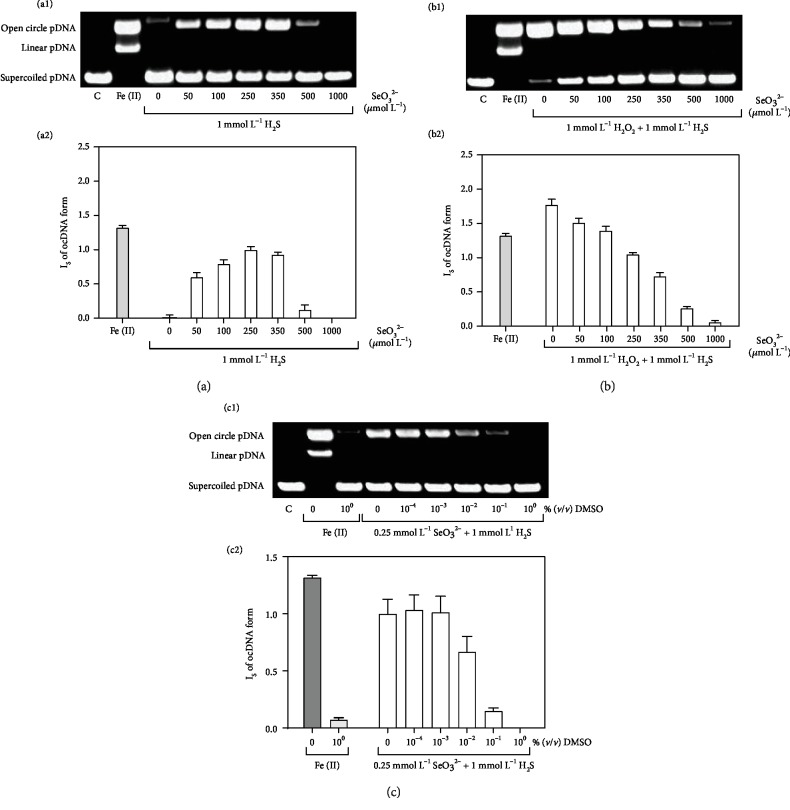
The influence of H_2_S/SeO_3_^2−^ on pDNA cleavage in the absence and presence of H_2_O_2_ and DMSO. Representative gels (a1, b1) and column graphs (a2, b2) indicating the effects of increasing concentrations of SeO_3_^2−^ (0-1 mmol L^−1^) on pDNA cleavage in the presence of 1 mmol L^−1^ H_2_S, without (a1, a2) and with 1 mmol L^−1^ H_2_O_2_ (b1, b2). The band at the bottom corresponds to the circular supercoiled form of pDNA, and the less intense band appearing above, in the case of Fe^2+^-H_2_O_2_, represents the linear form of pDNA. The top band corresponds to the nicked circular form of pDNA. The effects of 150 *μ*mol L^−1^ FeCl_2_ + 1 mmol L^−1^ H_2_O_2_ (full column) are shown for comparison. Values are the means ± S.E.M., *n* = 3. Representative gels (c1) and column graph (c2) showing the effects of increasing concentrations of DMSO on H_2_S/SeO_3_^2−^ (0.25/1 in mmol L^−1^)-induced pDNA cleavage. DMSO concentrations: 1 × 10^−4^% (*v*/*v*) DMSO = 14.1 *μ*mol L^−1^ DMSO; 1 × 10^−3^% = 141 *μ*mol L^−1^; 1 × 10^−2^% = 1.41 mmol L^−1^; 1 × 10^−1^% = 14.1 mmol L^−1^; 1 × 10^0^% = 141 mmol L^−1^. Values represent means ± S.E.M., *n* = 4.

It was of interest to know if the products of the H_2_S/SeO_3_^2−^ interaction have biological effects *in vitro*. Therefore, we investigated the direct effects of H_2_S and SeO_3_^2−^ on the cleavage of pDNA *in vitro* using the Fenton reaction as a positive benchmark control (Figure 6) [5].

Interestingly, neither H_2_S (1 mmol L^−1^) nor SeO_3_^2−^ (0-1 mmol L^−1^) alone significantly cleaved pDNA. In contrast, SeO_3_^2−^ in a concentration-dependent manner caused damage to DNA in the presence of 1 mmol L^−1^ H_2_S (Figures 6(a1) and 6(a2)). The observed cleavage of DNA caused by SeO_3_^2−^ and H_2_S showed a bell-shaped concentration ratio dependence similar to the one observed in the reduction of the ^·^cPTIO radical. We can suggest that the intermediate responsibility of the action can be a selenopolysulfide with a H_2_S : SeO_3_^2−^ ratio of 4 : 1. In the presence of H_2_O_2_, damage to pDNA by H_2_S occurs also without SeO_3_^2−^, since H_2_O_2_ now takes on the role of SeO_3_^2−^ as an oxidant, with the simultaneous formation of the ^·^OH radicals (Figures 6(b1) and 6(b2)). Further evidence for the involvement of radicals may come from the fact that dimethylsulfoxide (DMSO), a known ^·^OH scavenger [50, 51] frequently employed as solvent in biology, is able to interfere with the damage to DNA (Figures 6(c1) and 6(c2)).

### 3.4. H_2_S/SeO_3_^2−^ Modulates Tension of Isolated Thoracic Aorta

**Figure 7:**
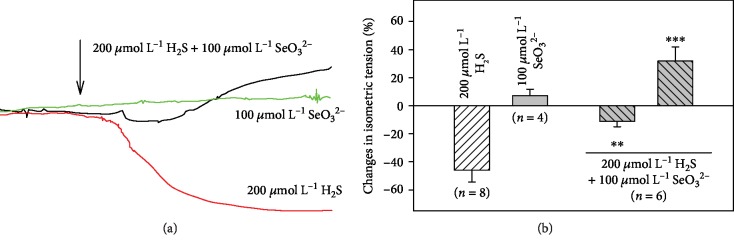
Time-dependent tonus of isolated thoracic aorta. The original records of changes in NA (1 *μ*mol L^−1^)-increased arterial tone of isolated rat thoracic aorta after addition of SeO_3_^2−^ (100 *μ*mol L^−1^), H_2_S (200 *μ*mol L^−1^), and H_2_S/SeO_3_^2−^ (200/100 in *μ*mol L^−1^) mixture. The arrow indicates the compound application (a). The effects of SeO_3_^2−^, H_2_S, and H_2_S/SeO_3_^2−^ on NA (1 *μ*mol L^−1^)-precontracted rings of rat thoracic aorta. The rings were exposed to bolus dose of H_2_S (200 *μ*mol L^−1^, relaxation), SeO_3_^2−^ (100 *μ*mol L^−1^, nonsignificant contraction), and that of the mixture H_2_S/SeO_3_^2−^ (100 *μ*mol L^−1^ of SeO_3_^2−^ immediately followed by 200 *μ*mol L^−1^ H_2_S). The SeO_3_^2−^/H_2_S mixture had a biphasic activity; firstly, it significantly relaxed the aorta, which was followed by significant contraction (b). Asterisks mark the statistical significance of H_2_S/SeO_3_^2−^ mixture vs. H_2_S (^∗∗^*P* < 0.01, ^∗∗∗^*P* < 0.001).

As some of our *in vitro* assays indicated an antioxidant activity of the H_2_S/SeO_3_^2−^ mixture, and H_2_S is known to promote relaxation of blood vessels [52], the impact of the H_2_S/SeO_3_^2−^ mixture on the isolated thoracic aorta was examined. The thoracic aorta was precontracted by noradrenaline (NA) (1 *μ*mol L^−1^). After a stabile plateau of the contraction was reached (Figure 7(a)), SeO_3_^2−^ (100 *μ*mol L^–1^) showed only negligible activity, while Na_2_S (200 *μ*mol L^−1^) significantly relaxed the aortic rings, in agreement with our previous studies [38]. A simultaneous addition of SeO_3_^2−^ (100 *μ*mol L^−1^) and H_2_S (200 *μ*mol L^−1^) resulted once more in a biphasic activity profile, where a minor relaxation was noticed first, followed by a pronounced contraction (Figure 7). It is supposed that the contraction effect may result also from the antioxidant properties of the mixture, similarly as it has been reported for ascorbate [53].

### 3.5. H_2_S/SeO_3_^2−^ Modulates Rat Systolic and Pulse Blood Pressure

**Figure 8:**
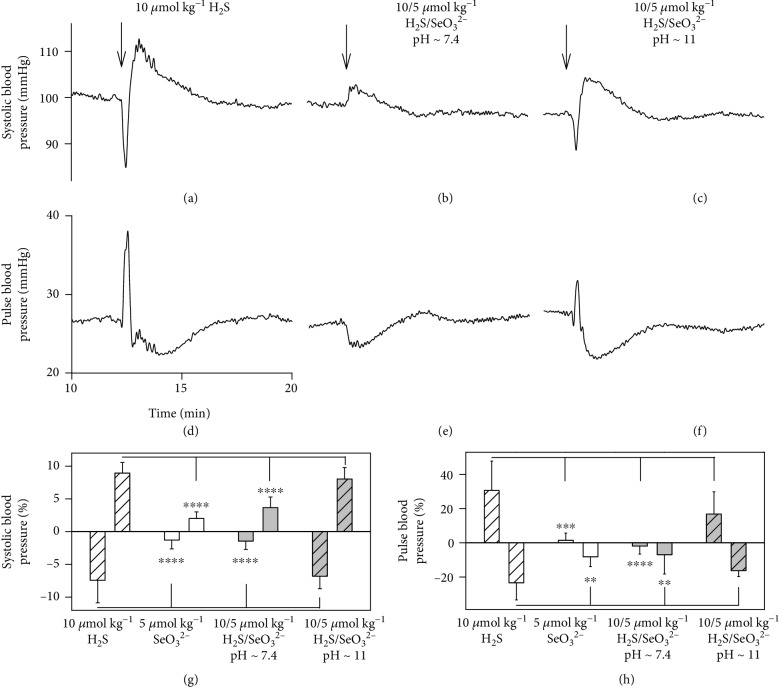
The time-dependent effects of SeO_3_^2−^, H_2_S, and H_2_S/SeO_3_^2−^ on rat BP and pulse BP. Representative traces of the time-dependent effect of i.v. bolus administration of H_2_S (10 *μ*mol kg^−1^; (a, d)) and its mixture with 5 *μ*mol kg^−1^ SeO_3_^2−^ prepared at pH ~ 7.4 (b, e) and pH ~ 11 (c, f) solution on BP (a, b, c) and pulse BP (d, e, f). Transient changes of rat BP (g) and pulse BP (h) after i.v. bolus administration of SeO_3_^2−^ (5 *μ*mol kg^−1^, empty column), H_2_S (10 *μ*mol kg^−1^, empty coarse column) and their mixture (SeO_3_^2−^/H_2_S, 5/10 in *μ*mol kg^−1^) prepared at pH ~ 7.4 (grey column) and pH ~ 11 (grey coarse column). Data are presented as means ± SD; *n* = 5‐10. To test a statistical significance between group differences, we used one-way ANOVA followed by Dunnett's test for multiple comparisons. Hence, we also observed the biphasic effect of Na_2_S in our previous study [54]; we compared a set of “first part” and “second part” effects of SeO_3_^2−^, H_2_S/SeO_3_^2−^ at pH ~ 7.4, and H_2_S/SeO_3_^2−^ at pH ~ 11.0 to the corresponding effect of Na_2_S on systolic or pulse blood pressure. Only the mixture of H_2_S/SeO_3_^2−^ prepared at pH ~ 11.0 was able to generate similar decrease and subsequent increase or *vice versa* in systolic blood pressure or pulse blood pressure as the H_2_S, respectively. Asterisks mark the statistical significance as follows: ^∗∗^*P* < 0.01, ^∗∗∗^*P* < 0.001, and ^∗∗∗∗^*P* < 0.0001.

Since the products of the H_2_S/SeO_3_^2−^ interaction modulated the tension of the thoracic aorta, we subsequently studied whether the products influence blood pressure (BP). Intravenous (i.v.) administration of 5 *μ*mol kg^−1^ SeO_3_^2−^ had only minor effects on BP (Figure 8(g)). The administration of 10 *μ*mol kg^−1^ of Na_2_S transiently decreased and increased BP (Figures 8(a) and 8(g)), as observed in our previous study [54]. The stock solution of the H_2_S/SeO_3_^2−^ mixture (20/10 in mmol L^−1^) prepared in 0.9% NaCl was colorless and had pH ~ 11. However, when the mixture was prepared in solution with pH 7.4, it had orange color with an absorption maximum at 570 nm ([Supplementary-material supplementary-material-1]), indicating formation of the sulfur-selenium complexes [40]. The i.v. administration of the mixture H_2_S/SeO_3_^2−^ (10/5 *μ*mol kg^−1^, pH ~ 7.4), in comparison to H_2_S alone, inhibited both BP decrease and increase (Figures 8(b) and 8(g)). The effects of the mixture were less pronounced at pH ~ 11, being the effects at this pH similar to those observed for H_2_S alone (Figures 8(c) and 8(g)).

The studied compounds influenced pulse BP, as an important parameter of cardiovascular system reflecting arterial stiffness [55, 56]. The administration of 5 *μ*mol kg^−1^ SeO_3_^2−^ intravenously had minor effects on pulse BP (Figure 8(h)). The administration of 10 *μ*mol kg^−1^ of Na_2_S transiently increased and later decreased pulse BP (Figures 8(d) and 8(h)) [54]. The i.v. administration of the mixture H_2_S/SeO_3_^2−^ (10/5 in *μ*mol kg^−1^, pH ~7.4), with comparison to H_2_S alone, eliminated pulse BP increase, but did not affect pulse BP decrease (Figures 8(e) and 8(h)). The effects of the mixture were less pronounced at pH ~ 11 and were similar to those observed for H_2_S alone (Figures 8(f) and 8(h)). The inefficiency of the H_2_S/SeO_3_^2−^ products at pH ~ 11 may be connected with the minor effect of the mixture on the ^·^cPTIO radical reduction at high pH (Figures 3(e) and 3(f)). This may imply that the reduction properties of the mixture could be responsible for their effects on systolic and pulse BP. The results confirm that the products of the H_2_S/SeO_3_^2−^ interaction depend on pH and influence differently the cardiovascular system. In conclusion, the reactivity and biological activity of the H_2_S/SeO_3_^2−^ interaction products prepared at pH 7.4 differ from those of Na_2_S alone.

## 4. Discussion and Conclusions

Overall, our studies demonstrate that the two suspected and commonly used “antioxidants,” H_2_S and SeO_3_^2−^, are not necessarily typical reducing agents, such as ascorbic acid or tocopherol, when employed on their own. Interestingly, these two chalcogen agents, when added together, rapidly activate each other and form a cascade of considerably more reactive, often reducing species, supposing the involvement of inorganic HSSeSH and polysulfides S*_x_*^2−^, which may account for some of the observed biological actions. The nature of some of these intermediate reactive sulfur and/or selenium species was suggested in a recently published review [40].

The fast and efficient reduction of the ^·^cPTIO radical by H_2_S/SeO_3_^2−^ products (Figures 1 and 4) supports the notion that the initial intermediate(s) formed by the reaction of H_2_S with SeO_3_^2−^ are responsible for this kind of action. The bell shape and the maximum radical scavenging activity of H_2_S : SeO_3_^2−^ at a ratio of ~4 : 1 (Figure 2) may indicate the suggested formation of (HSS)_2_Se [40]. The kinetics and efficiency of the H_2_S/SeO_3_^2−^ products to reduce ^·^cPTIO (Figure 3) point out to complex pH-dependent chemical and radical reactions of the species.

Reactions of H_2_S or polysulfides with SeO_3_^2−^ and/or SeCl_4_ were reported [40, 57–59]. They point towards a rapid conversion of SeO_3_^2−^ and SeCl_4_ to an intermediate, probably HSSeSH, and a subsequent, slower reductive elimination of this intermediate to elemental (mixed) chalcogen particles and disulfides [40]. However, to our knowledge, there is no information about the formation and detection of HSSeSH in cells or its cytoprotective or other biological effect. The first synthesis of H-S-Se-S-H (1.3-dithiatriselane) was reported by Hahn and Klünsch in 1994, but the stability and reactivity in aqueous solution were not investigated. Solid HSSeSH has a melting point at −40°C. It was one component of a mixture of H_2_S_2_Se_n_, prepared by the interaction of 2 H_2_S with Se_2_Cl_2_ [60].

The EPR data (Figure 5) once more confirm that SeO_3_^2−^ on its own is not an antioxidant; it becomes activated by *reduction*, for instance, by H_2_S, which concurrently is activated by *oxidation*. The mutual redox activation is fast, and, as in the case of the ^·^cPTIO radical scavenging, the pristine mixture of H_2_S and SeO_3_^2−^ is most active, with a decrease of activity over the time, pointing once more at simple H_2_S*_x_* or H_2_S*_x_*Se, and notably HSSeSH, but not an S*_x_*Se*_y_*, as being responsible for this kind of activity.

We found that the products of this described H_2_S/SeO_3_^2−^ interaction have several noteworthy biological effects, involving ROS scavenging, modulation of the redox state, reaction with DNA, tensing isolated aorta, and influencing BP and pulse BP (Figures 6–8). These effects obviously need to be investigated further and in considerably more detail and were not present or were less pronounced when H_2_S or selenite acted alone. The properties of the products of the H_2_S/SeO_3_^2−^ interactions significantly depended on the H_2_S/SeO_3_^2−^ molar ratio, pH, and preincubation time. The combination of these variables makes the work with H_2_S/SeO_3_^2−^ very complicated, and these facts should be taken into account at the time of designing *in vitro* and *in vivo* experiments. These properties of the products may explain the previously published beneficial and contrasting toxic Se effects, for example, in conditions of oxidative stress and cancer [12–14, 18, 20, 22, 23, 30].

H_2_S is endogenously produced *in vivo* in most, if not in all, cells, and H_2_S donors are commonly used in biological experiments, and they are considered to be applied in medicine. Our results suggest that in biological experiments with selenite, in its nutrition supplement and clinical use, effects of the H_2_S/SeO_3_^2−^ interaction should be considered. While SeO_3_^2−^ is used widely as a nutritional supplement already, one may, in the future, wish to spice it up with some reduced sulfur. Natural spices such as garlic and onions contain suitable sulfide releasing agents, such as diallyltrisulfide (DATS) and diallyltetrasulfide (DATTS), which both occur naturally in garlic, or dipropyltrisulfide and dipropyltetrasulfide, both present in onions [61]. Our results imply that application research of suitable H_2_S/SeO_3_^2−^ supplements may lead to the beneficial effects in pathological conditions arising, e.g., from ROS overproduction.

## Data Availability

All findings and conclusions are based on the presented figures in the main text or in the supplementary information. Original source files (UV-VIS spectra, EPR spectra, DNA gels, rat blood pressure records, and aorta relaxation records) can be sent from the corresponding author, Dr. Karol Ondrias, upon request.

## Abbreviations

ABS: Absorbance
BMPO: 5-*tert*-butoxycarbonyl-5-methyl-1-pyrroline-*N*-oxide
BP: Blood pressure
^·^cPTIO: 2-(4-Carboxyphenyl)-4,4,5,5-tetramethylimidazoline-1-oxyl-3-oxide
DTPA: Diethylenetriaminepentaacetic acid
EPR: Electron paramagnetic resonance
H_2_S: Hydrogen sulfide
H_2_O_2_: Hydrogen peroxide
i.p.: Intraperitoneal
i.v.: Intravenous
NA: Noradrenaline
pDNA: Plasmid DNA
SeCl_4_: Selenium tetrachloride
SeO_3_^2−^: Selenite
UV/VIS: Ultraviolet-visible.

## Supplementary Materials

Supplementary Materials The supplementary information's file (PDF) contains only additional figures with legends to the main manuscript text.
