# Supplementary material for: Products of Sulfide/Selenite Interaction Possess Antioxidant Properties, Scavenge Superoxide-Derived Radicals, React with DNA, and Modulate Blood Pressure and Tension of Isolated Thoracic Aorta
Source: Oxid Med Cell Longev. 2019 Nov 25;2019:9847650. doi: 10.1155/2019/9847650 (PMC6899273; doi:10.1155/2019/9847650)
Supplement: Supplementary Materials — The supplementary information's file (PDF) contains only additional figures with legends to the main manuscript text. [file 9847650.f1.pdf]

SUPPORTING INFORMATION

to

Products of sulfide/selenite interaction possess antioxidant properties, scavenge superoxide-derived radicals, react with DNA, modulate blood pressure and tension of isolated thoracic aorta

Marian Grman[1,*], Anton Misak[1,*], Lucia Kurakova[2], Vlasta Brezova[3], Sona Cacanyiova[4], Andrea Berenyiova[4], Peter Balis[4], Lenka Tomasova[1], Ammar Kharma[1], Enrique Domínguez-Álvarez[5], Miroslav Chovanec[6,†], Karol Ondrias[1,†]

[1] Institute of Clinical and Translational Research, Biomedical Research Center, Slovak Academy of Sciences, Dubravska cesta 9, 845 05 Bratislava, Slovak Republic
[2] Department of Pharmacology and Toxicology, Faculty of Pharmacy, Comenius University, Odbojarov 10, 832 32 Bratislava, Slovak Republic
[3] Faculty of Chemical and Food Technology, Slovak University of Technology, Radlinskeho 9, 812 37 Bratislava, Slovak Republic
[4] Institute of Normal and Pathological Physiology, Centre of Experimental Medicine, Slovak Academy of Sciences, Dubravska cesta 9, 841 04 Bratislava, Slovak Republic
[5] Instituto de Química Orgánica General, Consejo Superior de Investigaciones Científicas (IQOG-CSIC), 28006 Madrid, Spain
[6] Cancer Research Institute, Biomedical Research Center, Slovak Academy of Sciences, Dubravska cesta 9, 845 05 Bratislava, Slovak Republic

[*]Authors contributed equally to this work.
[†]Shared senior authorship.

Correspondence should be addressed to Karol Ondrias; karol.ondrias@savba.sk and Miroslav Chovanec; miroslav.chovanec@savba.sk

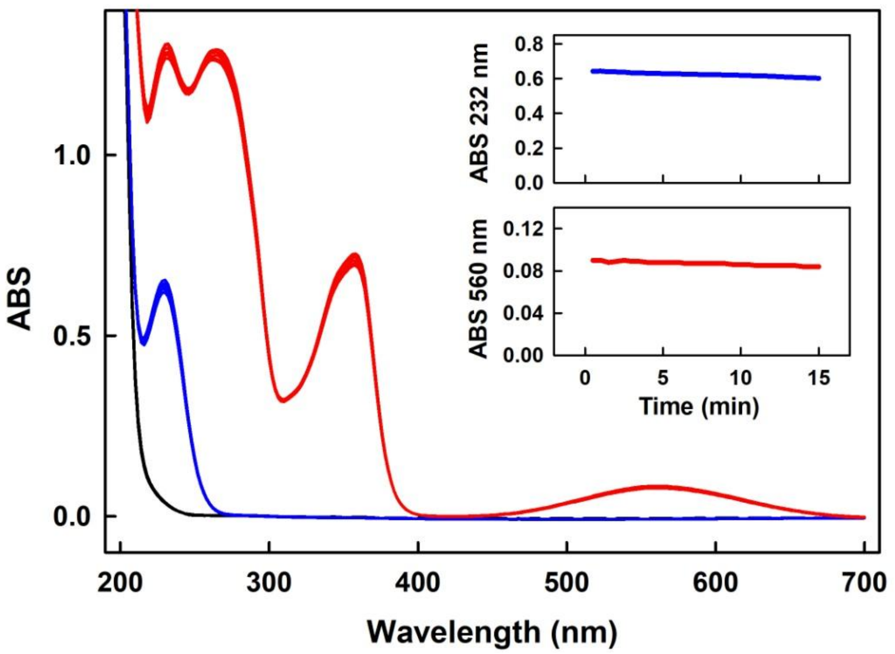

Supplementary Figure 1: Time-resolved UV-VIS spectra of Na_2SeO_4, H_2S and their mixture with $^{\bullet}$cPTIO. UV-VIS spectra of 100 µmol L^{-1} Na_2SeO_4 (repeated 3 x 30 s, black), subsequent addition of 100 µmol L^{-1} H_2S (30 x 30 s, blue) followed by 100 µmol L^{-1} $^{\bullet}$cPTIO (30 x 30 s, red). Upper insert: Kinetic trace of ABS at 232 nm (corresponding to HS^{-}) in the mixture of 100 µmol L^{-1} Na_2SeO_4 + 100 µmol L^{-1} H_2S (blue). Lower insert: Kinetic trace of 100 µmol L^{-1} $^{\bullet}$cPTIO reduction in the mixture of 100 µmol L^{-1} Na_2SeO_4 + 100 µmol L^{-1} H_2S (red) measured as change of ABS at 560 nm. Buffer: 100 mmol L^{-1} sodium phosphate buffer, 100 µmol L^{-1} DTPA, pH 7.4 at 37 °C.

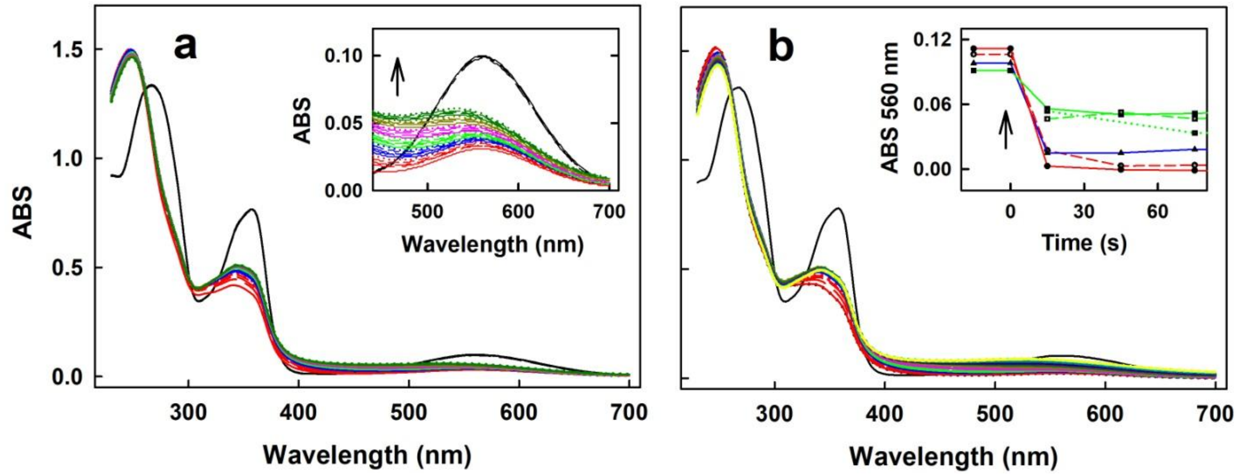

Supplementary Figure 2: Time resolved UV-VIS spectra of studied compounds.

(a) Time resolved UV-VIS spectra of $^{\bullet}$cPTIO, $SeCl_4$ and their mixture with H_2S. UV-VIS spectra of 100 μmol L^{-1} $^{\bullet}$cPTIO with 100 μmol L^{-1} $SeCl_4$ (repeated 3 times every 30 s, black). Subsequent addition of 100 μmol L^{-1} H_2S measured every 30 s for 15 min. Inset represents detail of ABS at 440-710 nm. The arrow indicates the increase of absorbance at around 420 nm.

(b)Time resolved UV-VIS spectra of the interaction of $^{\bullet}$cPTIO/SeO_3^{2-} with H_2S in argon de-aerated solutions. UV-VIS spectra of 100 μmol L^{-1} $^{\bullet}$cPTIO with 100 μmol L^{-1} SeO_3^{2-} (black) and subsequent addition of 100 μmol L^{-1} H_2S (spectra were collected every 30 s for 30 min, the first spectrum, indicated by the red line, was measured in 15th second after addition of H_2S). Inset: The reduction of the $^{\bullet}$cPTIO radical was detected as the decrease of ABS at 560 nm. Red lines: 110 μmol L^{-1} H_2S was added to $^{\bullet}$cPTIO/SeO_3^{2-} (110/110 in μmol L^{-1}); Blue lines: 100 μmol L^{-1} H_2S was added to $^{\bullet}$cPTIO/SeO_3^{2-} (100/100 in μmol L^{-1}) and green lines: 60 μmol L^{-1} H_2S was added to $^{\bullet}$cPTIO/SeO_3^{2-} (100/100 in μmol L^{-1}). Arrow marks H_2S addition.

The solid red line of UV-VIS spectra indicates the first spectrum after addition of H_2S into $SeCl_4$ or SeO_3^{2-}, which is followed each 30 s by: long dash red, medium dash red, short dash red, dotted red, solid blue line, long dash blue, medium dash blue, *etc*. Buffer: 100 mmol L^{-1} sodium phosphate, 100 μmol L^{-1} DTPA, pH 7.4, 37 °C.

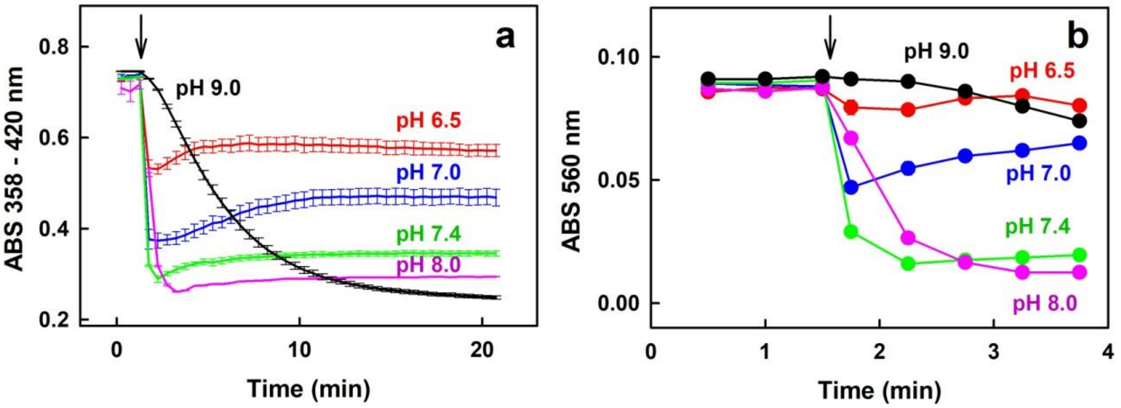

Supplementary Figure 3: Effect of pH on the kinetics of •cPTIO reduction induced by H_2S in the presence of SeO_3^{2-}.

The reduction of the •cPTIO radical was detected as decrease of ABS at 358 nm minus ABS at 420 nm (a) or as a decrese of the absorbance at 560 nm (b). Buffer: 100 mmol L^{-1} sodium phosphate, 100 µmol L^{-1} DTPA, pH 6.5, 7.0, 7.4, 8.0, and 9.0 at 37 °C. Arrow indicates addition of H_2S (100 µmol L^{-1}) into •cPTIO/SeO_3^{2-} (100/50 in µmol L^{-1}) solution. In the case of experiments at pH 6.5 and 7.0, UV-VIS spectra of H_2S/SeO_3^{2-} were subtracted from the •cPTIO/H_2S/SeO_3^{2-} spectra. Values are the means ± S.E.M., n = 2-3.

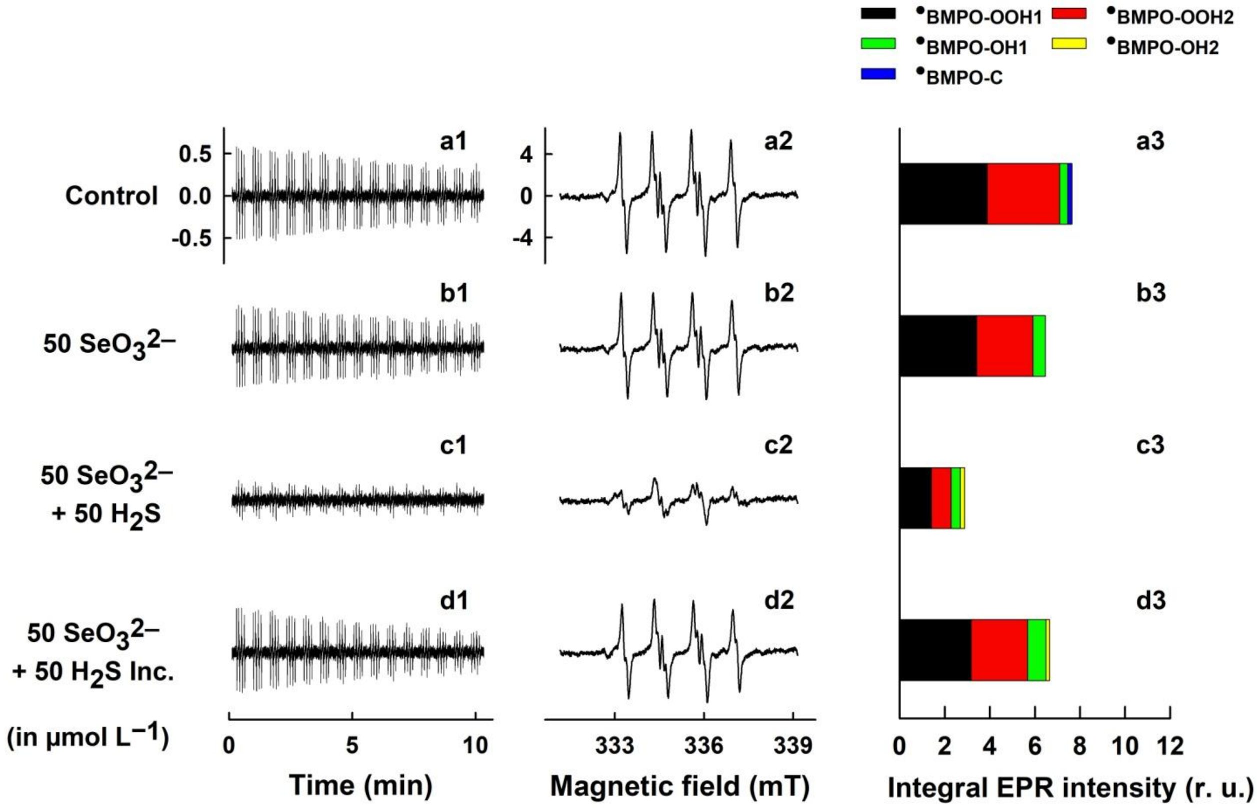

Supplementary Figure 4: EPR spectra of •BMPO in the presence of $O_2^{\bullet-}$ and modulated by H_2S/SeO_3^{2-}. Representative EPR spectra of the •BMPO-adducts monitored in 10% v/v saturated KO_2/DMSO solution in 50 mmol L^{-1} sodium phosphate buffer, 0.1 mmol L^{-1} DTPA, pH 7.4, 37 °C in the presence of studied compounds and 20 mmol L^{-1} BMPO. The sets of individual EPR spectra of the •BMPO-adducts monitored upon 15 sequential scans, each 42 s (a1-d1), starting acquisition 2 min after sample preparation in: control 10% v/v KO_2/DMSO in the buffer (a1), KO_2/DMSO in the presence of 50 µmol L^{-1} SeO_3^{2-} (b1), 50/50 in µmol L^{-1} H_2S/SeO_3^{2-} (c1) and 50/50 in µmol L^{-1} H_2S/SeO_3^{2-} preincubated 5 min before KO_2/DMSO addition (d1). The details of accumulated 15 spectra (a2-d2). The intensities of the time-resolved EPR spectra (a1-d1) and detailed spectra (a2-d2) are comparable, as they were measured at the identical EPR settings. EPR 0.1 mT modulation amplitude was used. (a3-d3) comparison of integral EPR spectra intensities of the individual components of simulated BMPO + $O_2^{\bullet-}$ without (control) and with chalcogen species showed in a1-d1. The first five EPR spectra were accumulated and used for simulation. The data represent the means of n = 2, standard error was ≤ 10 % of the mean value. The •BMPO-OOH and •BMPO-OH were simulated based on two conformers. The means of used hyperfine coupling constants were: •BMPO-OOH1 a_N = 13.30±0.03 G; a_H = 11.7±0.1 G; •BMPO-OOH2 a_N = 13.24±0.03 G; a_H = 9.4±0.1 G; •BMPO-OH1 a_N = 13.7±0.3 G; a_H = 12.3±0.4 G; •BMPO-OH2 a_N = 13.6±0.2 G; a_H = 15.3±0.1 G. For •BMPO-C a_N = 15.2±0.1 G; a_H = 21.5±0.1 G.

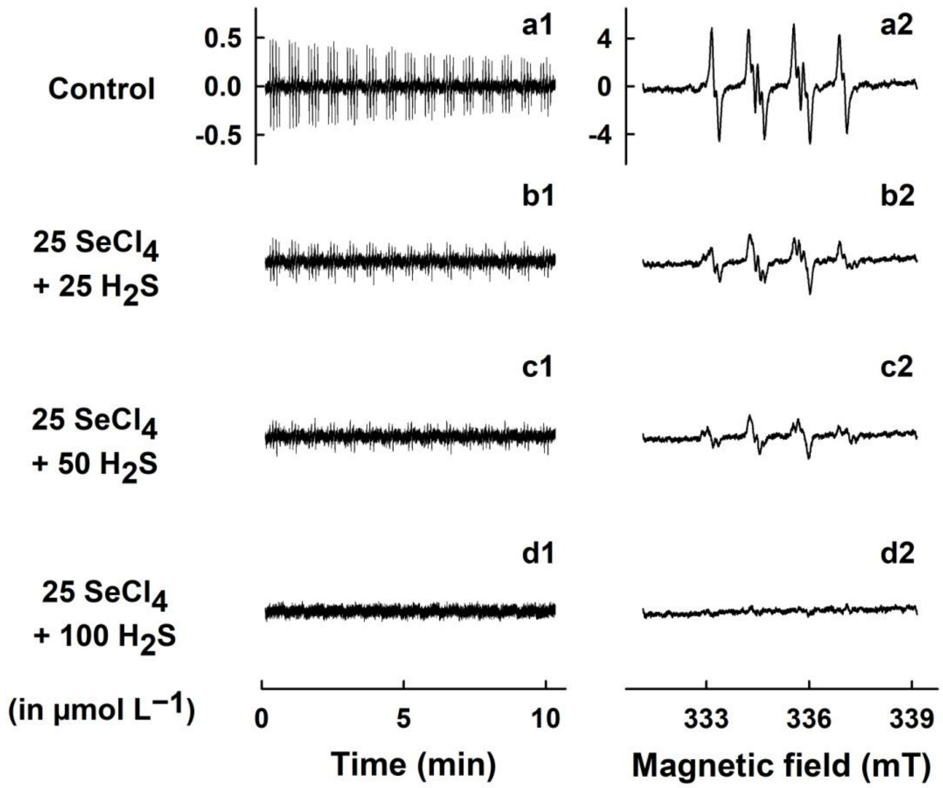

Supplementary Figure 5: EPR spectra of •BMPO in the presence of $O_2^{\bullet-}$ modulated by $H_2S/SeCl_4$.
Representative EPR spectra of the •BMPO-adducts monitored in 10% v/v saturated KO_2/DMSO solution in 50 mmol L^{-1} sodium phosphate buffer, 0.1 mmol L^{-1} DTPA, pH 7.4, 37 °C in the presence of studied compounds and 20 mmol L^{-1} BMPO. The sets of individual EPR spectra of the •BMPO-adducts monitored upon 15 sequential scans, each 42 s (a1-d1), starting acquisition 2 min after sample preparation in: control 10% v/v KO_2/DMSO in the buffer (a1), KO_2/DMSO in the presence of 25/25 in µmol L^{-1} $H_2S/SeCl_4$ (b1), 50/25 in µmol L^{-1} $H_2S/SeCl_4$ (c1) and 100/25 in µmol L^{-1} $H_2S/SeCl_4$ (d1). The detail of accumulated 15 spectra (a2-d2). The intensities of the time-resolved EPR spectra (a1-d1) and detailed spectra (a2-d2) are comparable, as they were measured at the identical EPR settings. EPR modulation amplitude was 0.1 mT.

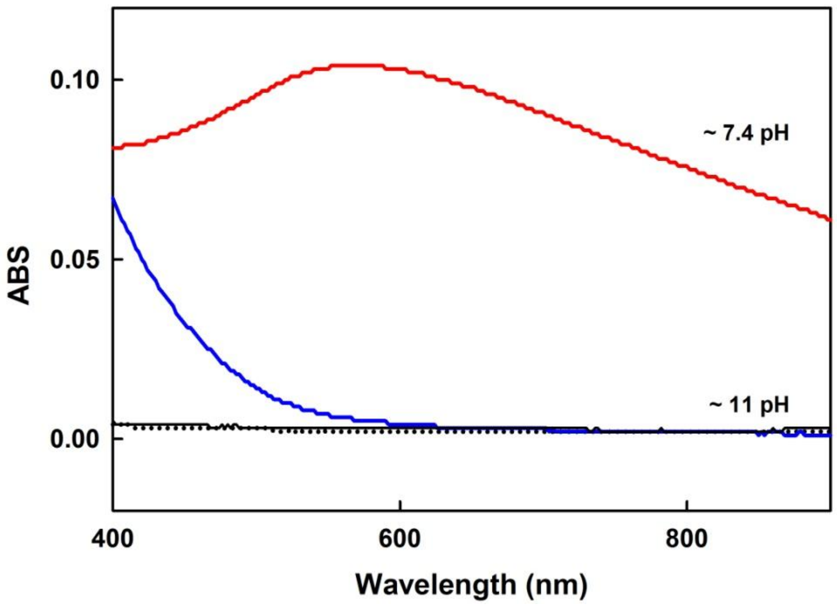

Supplementary Figure 6: VIS spectra of H_2S, SeO_3^{2-} and their mixture.
H_2S (100 µmol L^{-1}; black full), SeO_3^{2-} (50 µmol L^{-1}; dotted black) and their mixture (100/50 in µmol L^{-1}) prepared in 0.9 % NaCl pH ~ 11 (blue) and in phosphate buffer of pH ~ 7.4 (red). Sample with phosphate buffer was prepared as follows: 123.5 µL of 100 mmol L^{-1} phosphate buffer, 100 µmol L^{-1} DTPA, pH 7.4, 37°C; + 14 µL of 1 mol L^{-1} HCl; + 62.5 µL of 40 mmol L^{-1} SeO_3^{2-} prepared in 0.9 % NaCl and + 50 µL of 100 mol L^{-1} H_2S in H_2O. Final concentrations in the stock mixture of 250 µL volume were 20 mmol L^{-1} Na_2S and 10 mmol L^{-1} SeO_3^{2-}. pH was measured by paper pH indicator.
